# Hippocampal Volume and Plasma Brain-Derived Neurotrophic Factor Levels in Patients With Depression and Healthy Controls

**DOI:** 10.3389/fnmol.2022.857293

**Published:** 2022-05-06

**Authors:** Rintaro Fujii, Keita Watanabe, Naomichi Okamoto, Tomoya Natsuyama, Hirofumi Tesen, Ryohei Igata, Yuki Konishi, Atsuko Ikenouchi, Shingo Kakeda, Reiji Yoshimura

**Affiliations:** ^1^Department of Psychiatry, University of Occupational and Environmental Health, Kitakyushu, Japan; ^2^Open Innovation Institute, Kyoto University, Kyoto, Japan; ^3^Department of Radiology, Graduate School of Medicine, Hirosaki University, Hirosaki, Japan

**Keywords:** hippocampus, subregion, volume, brain-derived neurotrophic factor, major depression

## Abstract

The aim of the present study was to investigate associations between hippocampal subfield volumes and plasma levels of brain-derived neurotrophic factor (BDNF) in patients experiencing a first episode of major depression (MD) (*n* = 30) as compared to healthy controls (HC) (*n* = 49). Covariate-adjusted linear regression was performed to compare the MD and healthy groups, adjusting for age, sex, and total estimated intracranial volume. We demonstrated that there were no differences in total hippocampal volume between the MD and HC groups. However, the volumes of the hippocampus-amygdala-transition-area (HATA) on the left side of the brain as well as the parasubiculum, presubiculum, and fimbria on the right side were statistically significantly smaller in the MD group than in the HC group. Furthermore, the volume of the hippocampal fissure on the right side was statistically significantly smaller in the HC group than in the MD group. In the MD group, we found a positive linear correlation between hippocampal volume and plasma BDNF concentrations in the CA4 area on the left side (*p* = 0.043). In contrast, in the HC group, we found a negative linear correlation between parasubiculum volume on the right side and plasma BDNF concentrations (*p* = 0.04). These results suggest that some hippocampal subfields may already be atrophic at the start of MD. In addition, our findings suggest that the sensitivity of the right parasubiculum region to BDNF may differ between MD and HC groups. These findings guide future research directions and, if confirmed, may ultimately inform medical guidelines.

## Introduction

Depression is one of the most commonly occurring mental disorders, with a total lifetime prevalence of approximately 15–18%. Depression has a significant impact on patients’ quality of life, including social connectivity, economic security, and professional attainment (Malhi and Mann, 2018).

A previous report from the ENIGMA study, a comprehensive investigation conducted using Magnetic Resonance Imaging (MRI) to evaluate brain structure, function, and disease, demonstrated that hippocampal and amygdala volumes were statistically significantly decreased in depressed patients as compared to healthy controls (Schmaal et al., 2016). Chronic stress conditions are known to increase cortisol levels, and prior research suggests that this excessively increased cortisol may be associated with atrophy of the hippocampus and amygdala (Wingenfeld and Wolf, 2014).

BDNF (brain-derived neurotrophic factor), one of several neurotrophic factors that are widely present in the brain, promotes neurogenesis and synapse formation and is involved in synaptic plasticity (von Bohlen Und Halbachand von Bohlen Und Halbach, 2018). In stress-associated diseases, including depression, BDNF expression is decreased mainly in the hippocampus (Notaras and van den Buuse, 2020). Moreover, antidepressants (Jiang et al., 2019) and electroconvulsive therapy (Holtzmann et al., 2007) increase BDNF expression in the hippocampus, serum and plasma BDNF levels are decreased in depressed patients (Bocchio-Chiavetto et al., 2010), and antidepressant treatment increases BDNF levels as depressive symptoms are restored (Sen et al., 2008; Zhou et al., 2017). In addition, since BDNF crosses the blood-brain barrier (Pan et al., 1998), BDNF in the brain and periphery may affect hippocampal volume. For example, a recent study examining the relationship between hippocampal volume and serum BDNF concentrations in healthy controls found no relationship between the hippocampal volumes and serum BDNF concentrations (Puhlmann et al., 2021). From these findings into account, we hypothesized that hippocampal volume would be decreased in patients with MD as compared with healthy controls, and that BDNF concentrations would be positively correlated with hippocampal volumes.

The aim of this study was to investigate the relationship between hippocampal subregions and plasma BDNF concentrations, and to compare results between depressed patients and healthy controls. To the best of our knowledge, this is the first study to examine the relationship between hippocampal subregions and plasma BDNF levels in depressed and healthy controls.

## Materials and Methods

### Participants

We recruited patients with major depression (MD) and healthy controls (HC) as follows. Depressed patients were recruited from among consecutively presenting patients at the University hospital of the University of Occupational and Environmental Health, Japan. All MD patients were diagnosed by board-certified psychiatry based on standard criteria delineated in the Diagnostic and Statistical Manual for Mental Disorders-5 (DSM-5). We enrolled depressed patients who had not yet started antidepressants to remove the effects of medication of these associations. The control group was recruited from a neighboring community through posting or calls. Subjects enrolled in the HC group had never been diagnosed with any psychiatric disorder according to the findings of the Structured Clinical Interview for DSM Disorders (SCID). Written informed consent was obtained from all patients prior to participation. This study was approved by the ethics review board affiliated with our hospital and was conducted in accordance with the principles of the Declaration of Helsinki.

### Plasma Brain-Derived Neurotrophic Factor Levels

Plasma was collected from MD patients and HC in order to measure plasma BDNF; 15 mL of venous blood was collected in EDTA-2Na spray-coated blood vacutainers from each study participant, whole blood samples were centrifuged at 1,650 g for 25 min at room temperature. Subsequently, plasma samples were stored at −80°C. Plasma BDNF concentrations were measured using the BDNF Emax Immunoassay Kit (Promega, Madison, WI, United States), in which 96-well microplates were coated with anti-BDNF monoclonal antibody and incubated at 4°C for 18 h. The plates were incubated in a blocking buffer for 1 h at room temperature, and 100-fold diluted samples (i.e., diluted with assay buffer and BDNF standards) were kept at room temperature for 2 h under conditions of horizontal shaking and were then washed with the assay buffer. The plate was also washed with the buffer. Following this, the plate was incubated with anti-human BDNF polyclonal antibody for 2 h at room temperature and was then washed with the buffer again. The plates were incubated with anti-IgY antibody conjugated to horseradish peroxidase for 1 h at room temperature to induce a color reaction with a peroxidase substrate and a tetramethylbenzidine solution. The reaction was carried out in a 1 mol/L salt solution. The reaction was stopped with 1 mol/L hydrochloric acid and the absorbance at 450 nm was measured with an Emax automated microplate reader (Promega).

### Magnetic Resonance Imaging

MRI data were acquired using a 3T MR system (Signa EXCITE 3T; GE Healthcare, Waukesha, WI, United States) equipped with an eight-channel brain phased-array coil. Images were acquired via 3D fast-spoiled gradient recalled acquisition (3D-FSPGR). The acquisition parameters were as follows: repetition time/echo time, 10/4.1 ms; flip angle, 10°; field of view, 24 cm; resolution, 0.9 × 0.9 × 1.2 mm. All images were corrected for image distortion due to gradient non-linearity using the “Grad Warp” software program (Jovicich et al., 2006). The intensity inhomogeneity of the images was corrected using the “N3” function (Sled et al., 1998).

### Volume Measurement of the Hippocampal Subregion

**FIGURE 1:**
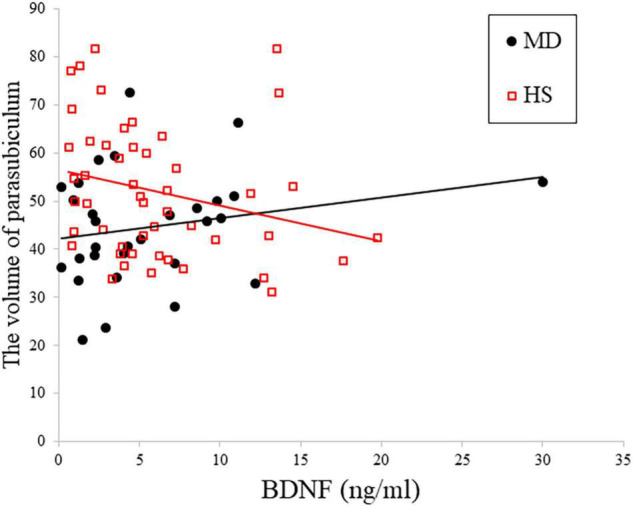
Relationship between plasma BDNF and depression in right parasubiculam adjusted for age, sex, and total intracranial volume. The interaction effects on the right parasubiculam were statistically significant (*p* < 0.05).

FreeSurfer open source software (v7.1.1)^1^ (Fischl, 2012) was used to evaluate hippocampal subregion volumes. The technical details of the cortical thickness analysis have been described elsewhere (Dale et al., 1999). In this study, hippocampal subregions were delineated using the Bayesian inference approach and the novel atlas algorithm for hippocampal morphology, which was constructed based on ultra-high-resolution *ex vivo* MRI data derived from autopsy brains (Iglesias et al., 2015). The results are shown in Figure 1. These results were used to classify the subiculum, presubiculum, parasubiculum, Cornu Ammonis (CA)1, CA3, CA4, molecular layer, hippocampus-amygdala-transition-area (HATA), fimbria, hippocampal tail, hippocampal fissure, and the whole hippocampus in terms of volumes. The left and right substructures were analyzed separately. Total hippocampal volume was calculated by summing the volumes of all hippocampal subregions other than the hippocampal fissure. The estimated intracranial volume was calculated using aseg segmentation (i.e., automatic subcortical segmentation of brain volume (Iglesias et al., 2015).

### Statistical Analysis

We compared depression and HC groups using chi-square tests for sex and the independent sample *t*-test for age, plasma BDNF levels, the estimated intracranial volume, total hippocampal volume, and each hippocampal subregion volume. Multiple regression analyses were performed to examine associations between plasma BDNF levels and hippocampal subregion volumes. For each hippocampal subregion, the correlation with plasma BDNF levels was analyzed. Covariates were adjusted for age, sex, and estimated intracranial volume. We evaluated the partial regression coefficients (β) and multiple correlation coefficients (r). In the hippocampal subregion that showed a significant correlation, the association with plasma BDNF levels was evaluated by partial correlation analysis (partial correlation coefficients: r’), excluding the effects of age, sex, and estimated intracranial volume. To assess the effects of the presence or absence of depression on plasma BDNF in each hippocampal subregion, we examined multiplicative interactions between MD and BDNF in a linear regression model. *P*-values (two-tailed) less than 0.05 were considered statistically significant. All statistical analyses were conducted using Stata statistical software (v. 17, StataCorp LLC, College Station, TX, United States).

## Results

Thirty patients with first-episode untreated MD and 49 HC were enrolled in the current study. The main background factors are shown in Table 1. There were no statistically significant differences in sex, age, or plasma BDNF concentrations between the HC and depression groups (sex: *p* = 0.12, age: *p* = 0.14, plasma BDNF: *p* = 0.79).

**TABLE 1:** Background factors in MD and healthy controls.

	MD	HC	
	*n* = 30	*n* = 49	*p*-value
Sex, male (%)	17 (57%)	36 (73%)	0.12
Age, mean (SD)	44.93 (12.97)	40.82 (11.37)	0.14
Plasma BDNF (ng/mL), mean (SD)	5.63 (5.86)	5.94 (4.71)	0.79
HDRS, mean (SD)	21.0 (5.97)	−	−
Duration of illness (month), median (range)	3 (1–10)	−	−

*HDRS, Hamilton Depression Rating Scale.*

The comparative volumes of each hippocampal subregion between the HC and MD groups using *t*-tests are shown in Table 2. The estimated intracranial volume was smaller in the MD group as compared to the HC group (*p* = 0.047), and there were no differences in total hippocampal volume between the MD and HC groups on either side of the brain. However, HATA volumes on the left side and parasubiculum, presubiculum, and fimbria volumes on the right side were statistically significantly smaller in the MD group as compared to the HC (HATA volumes on the left side: *p* = 0.02; parasubiculum, presubiculum, and fimbria volumes on the right side: *p* = 0.014, 0.005, 0.012). Furthermore, hippocampal fissure volumes on the right side were statistically significantly smaller in the HC group as compared to the MD (*p* = 0.035).

**TABLE 2:** A comparison between the MD Patients and healthy controls in nuclei of hippocampus.

	MD	HC	
	Mean (SD)	Mean (SD)	*p*-value
Estimated total intracranial volume	15,35,793 (135470.3)	16,01,980 (145165.9)	0.047*
**Left**			
Whole hippocampus	3224.07 (300.04)	3338.63 (319.02)	0.12
Parasubiculum	49.87 (10.83)	52.61 (9.70)	0.25
Presubiculum	270.65 (36.36)	286.35 (39.88)	0.083
Subiculum	429.08 (44.23)	439.35 (53.03)	0.38
CA1	600.11 (72.28)	622.16 (93.74)	0.27
CA3	198.73 (29.85)	203.53 (33.19)	0.52
CA4	505.95 (60.69)	524.96 (55.23)	0.16
Molecular layer	523.92 (50.85)	544.71 (57.81)	0.11
HATA	52.22 (9.73)	57.18 (8.57)	0.02*
Fimbria	93.50 (22.07)	101.56 (14.81)	0.056
Hippocampal tail	500.04 (82.58)	506.24 (75.70)	0.73
Hippocampal fissure	146.05 (20.81)	146.77 (30.15)	0.91
**Right**			
Whole hippocampus	3366.72 (335.78)	3481.48 (392.23)	0.88
Parasubiculum	44.52 (11.66)	52.05 (13.66)	0.014*
Presubiculum	253.86 (37.89)	280.07 (40.04)	0.005*
Subiculum	431.68 (53.73)	442.88 (54.33)	0.37
CA1	658.09 (67.79)	664.37 (103.25)	0.77
CA3	218.50 (33.25)	219.58 (33.49)	0.89
CA4	533.74 (59.61)	547.57 (60.56)	0.33
Molecular layer	553.09 (55.23)	568.06 (71.34)	0.33
HATA	55.15 (9.72)	56.91 (9.61)	0.44
Fimbria	88.29 (23.34)	101.32 (20.97)	0.012*
Hippocampal tail	529.79 (81.37)	548.70 (76.51)	0.3
Hippocampal fissure	169.34 (31.13)	155.22 (26.47)	0.035*

The volumes were normalized and given under: mean (SD), Age, sex, and estimated intracranial volume are covariates in analyses. *p < 0.05.

**FIGURE 2:**
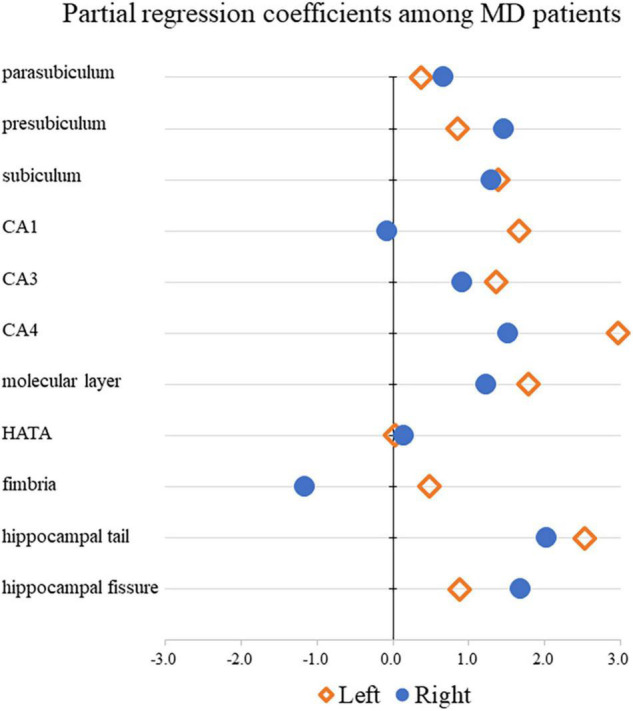
Comparison of partial regression coefficients for BDNF and hippocampal subregion volume ratio among MD patients.

**FIGURE 3:**
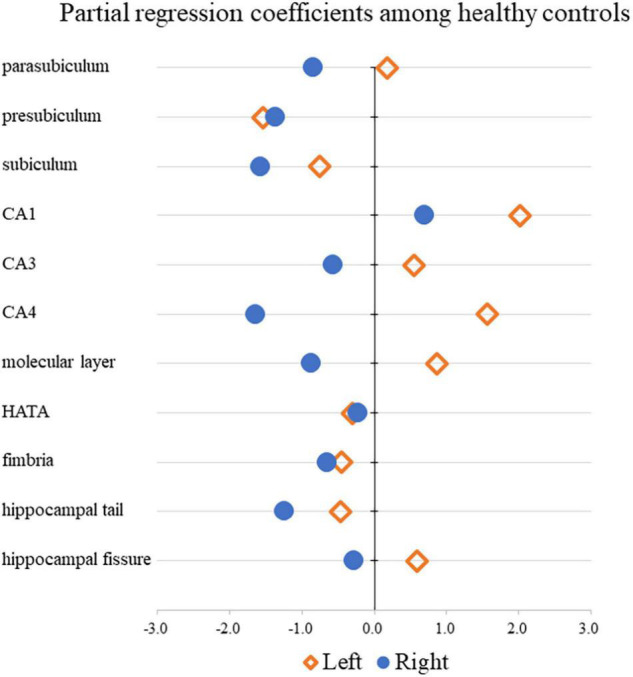
Comparison of partial regression coefficients for BDNF and hippocampal subregion volume ratio among HC.

Table 3 shows the results of multiple regression analyses evaluating associations between plasma BDNF concentrations and hippocampal subregional volumes. In the MD group, we found a positive linear correlation between hippocampal volume and plasma BDNF concentrations in the CA4 area on the left side [β = 2.96 (95%CI 0.10, 5.83), *r* = 0.77, *p* = 0.043]. In contrast, in the HS group, we found a negative linear correlation between plasma BDNF concentrations and parasubiculum volume on the right side [β = −0.85 (95%CI −1.66, −0.04), *r* = −0.42, *p* = 0.04] (Figure 1). Both also showed a significant correlation with plasma BDNF levels by partial correlation analysis (CA4 on the left side in the MD group: *r*’ = 0.39, *p* = 0.043; the parasubiculum on the right side in the HC group: *r*’ = −0.30, *p* = 0.04). The partial regression coefficients between plasma BDNF levels and hippocampal volumes calculated for each subregion showed different distributions in the MD and HC groups (Figures 2, 3). Figure 1 demonstrates the presence of an interaction between the right parasubiculum volume and plasma BDNF levels in the MD group (*p* = 0.013).

**TABLE 3:** Partial regression coefficient between BDNF and hippocampal subregion volume, and interaction between MD and plasma BDNF on hippocampal subregions.

	MD			HC			Interaction	
	β	95%CI	*p*-value	β	95%CI	*p*-value	F	*p*-value
**Left**								
Parasubiculum	0.38	−0.39, 1.14	0.321	0.17	−0.41, 0.75	0.557	0.29	0.589
Presubiculum	0.85	−1.76, 3.47	0.508	−1.54	−3.90, 0.83	0.197	1.99	0.164
Subiculum	1.38	−1.42, 4.18	0.319	−0.76	−3.61, 2.09	0.594	0.98	0.326
CA1	1.66	−2.49, 5.81	0.418	2.02	−3.33, 7.36	0.451	0.12	0.727
CA3	1.35	−0.03, 2.73	0.054	0.55	−1.32, 2.41	0.559	0.11	0.744
CA4	2.96	0.10, 5.83	0.043*	1.56	−1.10, 4.22	0.243	0.08	0.783
Molecular layer	1.78	−0.85, 4.42	0.175	0.87	−2.18, 3.91	0.568	0.02	0.883
HATA	0.03	−0.54, 0.60	0.917	−0.30	−0.80, 0.20	0.232	0.50	0.481
Fimbria	0.49	−1.01, 1.98	0.509	−0.46	−1.29, 0.38	0.277	1.06	0.308
Hippocampal tail	2.53	−2.76, 7.82	0.333	−0.47	−5.34, 4.39	0.846	0.26	0.614
Hippocampal fissure	0.88	−0.31, 2.06	0.139	0.59	−1.16, 2.34	0.501	0.04	0.852
**Right**								
Parasubiculum	0.66	−0.11, 1.43	0.089	−0.85	−1.66, −0.04	0.040*	6.50	0.013*
Presubiculum	1.46	−1.18, 4.10	0.267	−1.37	−3.79, 1.05	0.259	2.86	0.095
Subiculum	1.29	−2.13, 4.72	0.444	−1.58	−4.47, 1.31	0.277	1.93	0.169
CA1	−0.08	−4.08, 3.93	0.969	0.70	−4.74, 6.14	0.797	0.10	0.749
CA3	0.91	−0.73, 2.54	0.265	−0.58	−2.29, 1.14	0.501	0.72	0.395
CA4	1.52	−1.68, 4.71	0.338	−1.64	−4.51, 1.22	0.254	1.21	0.273
Molecular layer	1.23	−1.95, 4.41	0.432	−0.87	−4.52, 2.77	0.631	0.55	0.461
HATA	0.14	−0.47, 0.74	0.640	−0.23	−0.76, 0.29	0.375	0.41	0.527
Fimbria	−1.16	−2.63, 0.30	0.115	−0.66	−1.79, 0.48	0.249	0.59	0.443
Hippocampal tail	2.02	−3.13, 7.17	0.427	−1.24	−5.88, 3.40	0.592	0.88	0.351
Hippocampal fissure	1.68	−0.17, 3.53	0.074	−0.28	−1.85, 1.29	0.722	3.84	0.054

β is the partial regression coefficient. CI is the confidence interval. Age, sex, and all intracranial volume are covariates in analyses. The F-values represented the effect of MD and plasma BDNF on hippocampal subregions. *p < 0.05.

## Discussion

In this study, we found that the volumes of the left-sided HATA, the right-sided parasubiculum, the presubiculum, and the fimbria were statistically significantly smaller in first-episode untreated patients with MD as compared with a HC group. Results reported by Han et al. (2019) which evaluated the same (repetitive) MD group as well as patients on antidepressant medication, demonstrated that the MD group had statistically significantly smaller bilateral CA1, CA4, granule cell layer (GCL), molecular layer (ML), and whole hippocampal volumes as compared to the HC group (Han et al., 2019). Another reported that there were no statistically significant differences in the volume of any hippocampal subfield in the MD group (including cases who were or were not receiving medical treatment for depression) as compared with the HC group (Zhang et al., 2021). A prospective 7-year follow-up study of MD patients conducted by Wisse et al. (2015) found that the number of depressive episodes in the enrolled patients was associated with atrophy of the subiculum volume (Wisse et al., 2015; Zhang et al., 2021).

Moreover, in untreated women with MD, the volumes of the left subiculum, CA2-3, CA4, dentate gyrus, and right subiculum were statistically significantly smaller than those in the HC group in prior research (Han et al., 2016). We also note that 7T MRI was used by Brown et al. (2019) to examine the relationship between hippocampal subfield volumes and MD severity. These researchers found that right-sided CA1 and CA/43 volumes were negatively correlated with depression severity. In other words, the more severe the MD symptoms, the greater the atrophy of this hippocampal subfield (Brown et al., 2019). In light of the above results, it is likely that changes in the hippocampal subfield in MD patients are influenced by the number of depressive episodes, the presence or absence of medication, sex, and the severity of depression. However, our results suggest that some hippocampal subfields in the MD group may already be atrophic at the start of the disease.

BDNF is a neural-derived factor that is involved in neuroplasticity and is closely associated with neurogenesis and synaptic plasticity in the hippocampus. Prior studies conducted by this research group have reported that plasma and serum BDNF levels are statistically significantly lower in MD patients as compared with healthy controls (Kishi et al., 2018; Yoshimura et al., 2018). However, in the present study, no differences in plasma BDNF concentrations were observed between the MD and HC groups. The most likely reason for these discrepant findings is the small number of enrolled cases (given restrictions for medication use).

To the best of our knowledge, this is the first study to examine the relationship between plasma BDNF concentrations and hippocampal subfield volumes. A prior report examining hippocampal volumes and serum BDNF concentrations in first-episode, untreated MD patients demonstrated that there was a statistically significant positive correlation between hippocampal volume and serum BDNF concentrations in MD patients, whereas there was no correlation in the HC group (Eker et al., 2010). A report on elderly patients with MD also showed a statistically significant positive correlation between hippocampal volume and serum BDNF concentrations (Bouckaert et al., 2016). Although it is controversial whether serum or plasma BDNF concentrations reflect BDNF concentrations in the brain (i.e., whether serum or plasma BDNF is a valid biomarker for BDNF concentrations), BDNF is known to cross the blood-brain barrier. We conclude that there may be a relationship between low serum BDNF and hippocampal volume in patients with MD. However, the results of the present study demonstrate that there was no relationship between overall hippocampal volume and plasma BDNF concentrations in either the MD or the HC group. Nevertheless, in the MD group, we found a positive linear correlation between hippocampal volume and plasma BDNF concentrations in the CA4 region on the left side of the hippocampus, where neurogenesis is observed in neural adults (Kempermann et al., 2015; Gonçalves et al., 2016). It is interesting that a positive correlation was observed in this region, suggesting that BDNF levels may increase in the MD group to compensate for the atrophy in the left CA4 region. On the other hand, in the HC group, there was a negative linear correlation between the right-sided parasubiculum volume and plasma BDNF concentrations. Moreover, in the HC group, there was a negative linear correlation between the right-sided parasubiculum volume and plasma BDNF concentrations, and this region demonstrated an interaction between the MD and HC groups with respect to plasma BDNF concentrations. The interpretation of these results is unclear. However, we conclude that it is possible that the right parasubiculum region has different levels of sensitivity to BDNF when comparing patients with MD to healthy controls.

### Limitations

In addition to the substantial strengths of this study, we acknowledge several limitations. First, we conducted a cross-sectional study enrolling a small number of cases, and thus the causality of relationship between the observed hippocampal subfield changes and plasma BDNF levels is unclear. A longitudinal study enrolling a larger number of patients is needed to evaluate these findings more thoroughly and comprehensively. Future highly powered studies should also evaluate the effects of antidepressant medications on the observed findings.

## Conclusion

Differences in hippocampal subfield volume were observed when comparing enrolled first-episode untreated patients with MD to healthy controls. Some hippocampal subfields were correlated with plasma BDNF levels, and an interaction was observed between the right parasubiculum volume in the MD and HC groups with respect to plasma BDNF levels. These results suggest that some hippocampal subfields may already be atrophic at the start of MD, and that the sensitivity of the right parasubiculum region to BDNF may differ between MD and HC groups. Our findings guide future research directions and, if confirmed, may ultimately inform medical guidelines.

## Data Availability Statement

The original contributions presented in the study are included in the article/supplementary material, further inquiries can be directed to the corresponding author/s.

## Ethics Statement

The studies involving human participants were reviewed and approved by the University of Occupational and Environmental Health, Japan. The patients/participants provided their written informed consent to participate in this study.

## Author Contributions

RF, KW, NO, SK, and RY conceived and designed the experiments. RF, KW, SK, AI, NO, and RY performed the experiments. RF, KW, and NO analyzed the data. RF, KW, NO, and RY composed the manuscript. RF, KW, AI, TN, HT, YK, RI, SK, and RY provided expertise and edited the manuscript. All authors read the manuscript and are solely and jointly responsible for its content.

## Conflict of Interest

The authors declare that the research was conducted in the absence of any commercial or financial relationships that could be construed as a potential conflict of interest.

## Publisher’s Note

All claims expressed in this article are solely those of the authors and do not necessarily represent those of their affiliated organizations, or those of the publisher, the editors and the reviewers. Any product that may be evaluated in this article, or claim that may be made by its manufacturer, is not guaranteed or endorsed by the publisher.

## Footnotes

1
http://surfer.nmr.mgh.harvard.edu/
